# Human Contamination in Public Genome Assemblies

**DOI:** 10.1371/journal.pone.0162424

**Published:** 2016-09-09

**Authors:** Kirill Kryukov, Tadashi Imanishi

**Affiliations:** Department of Molecular Life Science, School of Medicine, Tokai University, Isehara, Kanagawa, Japan; Yeshiva University Albert Einstein College of Medicine, UNITED STATES

## Abstract

Contamination in genome assembly can lead to wrong or confusing results when using such genome as reference in sequence comparison. Although bacterial contamination is well known, the problem of human-originated contamination received little attention. In this study we surveyed 45,735 available genome assemblies for evidence of human contamination. We used lineage specificity to distinguish between contamination and conservation. We found that 154 genome assemblies contain fragments that with high confidence originate as contamination from human DNA. Majority of contaminating human sequences were present in the reference human genome assembly for over a decade. We recommend that existing contaminated genomes should be revised to remove contaminated sequence, and that new assemblies should be thoroughly checked for presence of human DNA before submitting them to public databases.

## Introduction

Databases of reference genome sequences is an important resource in vast number of biological and medical studies. E.g., in metagenomics a good reference of genome sequences is important. Contamination present in the reference genome sequence could lead to incorrect or confusing results [1]. The problem of contamination is known for over two decades [2]. Bacteria is the most common contaminant [3]. Human is another important source of contamination, since human is present at all stages of sample handling and lab procedures. Ancient DNA is particularly affected by human contamination [4]. However outside of the field of ancient DNA this problem receives little attention.

In previously study Longo et al. [5] investigated human contamination in non-primates by looking at SINE sequence in 2,749 genomes. In this study we set out to systematically survey available genome sequences for the evidence of contamination from non-repetitive human DNA. Ultraconserved sequences of 100% identity spanning over 200 bp are known to exist between human and mouse [6]. Therefore homology alone is not enough to conclude that contamination has occurred. In this study we used massive homology search and lineage specificity in order to discard the instances of true conservation and detect signals consistent with contamination. We were able to find numerous instances of sequence that can only be reasonably explained as contamination by human DNA.

## Results

We were able to detect 3,416 likely human originated sequences (LHO) within public genome sequences. Each of the LHO sequences is at least 100 bp long, and has strong similarity with human sequence (≥95% identity at the nucleotide level). Also, each LHO has homology within primates (other than human), that is much stronger than homology to any sequence outside primates (excluding the source genome of particular LHO sequence).

100 LHOs were found in non-primate mammals, 366 in non-mammal vertebrates, 2,792 in non-vertebrate eukaryotes, and 158 in prokaryotes (Tables 1–4; S1, S2, S3, and S4 Tables). In total 154 genomes were found to contain LHOs: 11 mammalian genomes, 15 non-mammal vertebrate genomes, 67 non-vertebrate eukaryote genomes and 61 prokaryote genomes.

**Table 1:** Mammalian genomes containing at least 2 kbp of likely human originated (LHO) sequence.

Organism	Assembly accession	Assembly date	Regions	Total LHO length (bp)	LHO bp / genome Mbp
*Felis catus*	GCF_000181335.2	2014-11-07	42	15,056	5.79
*Rattus norvegicus*	GCF_000001895.5	2014-07-01	24	5,907	2.16
*Bison bison bison*	GCF_000754665.1	2014-10-08	8	2,750	1.04

**Table 2:** Non-mammal vertebrate genomes containing at least 2 kbp of likely human originated (LHO) sequence.

Organism	Assembly accession	Assembly date	Regions	Total LHO length (bp)	LHO bp / genome Mbp
*Chelonia mydas*	GCF_000344595.1	2013-03-18	130	33,289	15.77
*Cathartes aura*	GCA_000699945.1	2014-06-11	57	14,273	12.50
*Serinus canaria*	GCF_000534875.1	2014-01-15	111	13,488	11.97
*Meleagris gallopavo*	GCF_000146605.2	2014-11-24	13	3,189	2.92
*Salmo salar*	GCA_000233375.4	2015-06-10	9	3,141	1.03
*Periophthalmus magnuspinnatus*	GCA_000787105.1	2014-12-02	13	2,359	3.43

**Table 3:** Non-vertebrate eukaryote genomes containing at least 2 kbp of likely human originated (LHO) sequence.

Organism	Assembly accession	Assembly date	Regions	Total LHO length (bp)	LHO bp / genome Mbp
*Plasmodium gaboni*	GCA_000576715.1	2014-02-03	1,404	335,303	20,566.80
*Lotus japonicus*	GCA_000181115.1	2008-06-27	576	150,537	1,018.43
*Phanerochaete chrysosporium RP-78*	GCA_000167175.1	2004-04-29	129	35,036	1,174.04
*Toxoplasma gondii COUG*	GCA_000338675.1	2013-02-07	127	26,468	415.54
*Lachancea waltii NCYC 2644*	GCA_000167115.1	2004-03-15	76	18,422	1,688.22
*Saccharomyces cerevisiae CBS 7960*	GCA_000192455.1	2011-03-18	60	11,195	916.47
*Toxoplasma gondii*	GCA_000256725.1	2012-03-27	53	9,963	158.19
*Hordeum vulgare subsp*. *vulgare*	GCA_001077415.1	2014-07-08	76	9,391	5.77
*Saccharomyces paradoxus NRRL Y-17217*	GCA_000166955.1	2003-03-28	34	7,498	631.54
*Sarcocystis neurona*	GCA_000875885.1	2015-02-13	20	6,350	53.96
*Pneumocystis jirovecii*	GCA_000333975.2	2012-12-19	19	6,145	751.22
*Saccharomyces mikatae IFO 1815*	GCA_000166975.1	2003-03-28	10	3,793	330.68
*Hordeum pubiflorum*	GCA_000582825.1	2013-08-30	19	3,607	2.55
*Chlamydomonas reinhardtii*	GCF_000002595.1	2007-10-15	11	3,505	33.25
*Zea mays*	GCF_000005005.1	2013-10-24	14	2,854	1.39
*Saccharina japonica*	GCA_000978595.1	2015-04-22	6	2,782	5.18
*Melampsora pinitorqua Mpini7*	GCA_000464645.1	2014-06-06	9	2,672	80.36
*Nematostella vectensis*	GCF_000209225.1	2007-08-22	10	2,567	8.63
*Toxoplasma gondii ME49*	GCA_000006565.2	2013-08-02	10	2,242	34.25
*Toxoplasma gondii TgCATBr5*	GCA_000259835.1	2011-08-26	10	2,085	33.83

**Table 4:** Prokaryote genomes containing at least 2 kbp of likely human originated (LHO) sequence.

Organism	Assembly accession	Assembly date	Regions	Total LHO length (bp)	LHO bp / genome Mbp
*Pseudomonas aeruginosa*	GCA_000825745.1	2014-12-01	34	7,301	1,146.80
*Gemmata obscuriglobus UQM 2246*	GCA_000171775.1	2007-10-23	10	4,221	460.72
*candidate division TM7 single-cell isolate TM7a*	GCA_000170635.1	2007-06-08	14	3,099	1,082.27
*Pseudomonas sp*. *2(2015)*	GCA_000955865.1	2015-03-18	5	2,946	503.02

We provide all LHO sequences in FASTA format in S1 Dataset, which contains the following files: “LHO-sequences-non-primate-mammals.fna”, “LHO-sequences-non-mammal-vertebrates.fna”, “LHO-sequences-non-vertebrate-eukaryotes.fna”, and “LHO-sequences-prokaryotes.fna”. The name of each LHO sequence consists of the original sequence names, followed by the 1-based coordinates of the LHO within that sequence, inclusive on both ends. We also provide the coordinates of LHO regions in BED format in S2 Dataset. The BED files allow masking the LHO regions in non-human genome using bedtools [7], with the following command: “bedtools maskfasta -fi genome.fa -bed lho.bed -fo masked.fa”.

Tables 1–4 list genomes containing over 2 Kbp of apparently human sequence. Among mammals, cat genome is the most contaminated, containing over 15 Kbp of human DNA. It is followed by rat and bison genomes. It’s surprising to see many LHO’s in the rat genome, as it has been studies and refined extensively and is now at build number 6.0.

In other vertebrates, the genome of green turtle (*Chelonia mydas*) is leading with 33 Kbp of human sequence. Next are the two bird genomes: turkey vulture (*Cathartes aura*), and canary (*Serinus canaria*), with 14 and 13 Kbp of human sequence, respectively.

Among non-vertebrate eukaryotes we find the most contaminated genome: *Plasmodium gaboni* (GenBank accession GCA_000576715.1)–its content of human DNA is extremely high at 335 Kbp. More than 2% of this assembly consists of apparently human DNA. Since *Plasmodium* is a known human parasite, the one possible explanation for this contamination is *Plasmodium* sample was not completely separated from source human tissue before sequencing.

The next notably contaminated genome is *Lotus japonicus*, with ~151 Kbp of human sequence. Over 80% of all LHO sequences we detected are in non-vertebrate eukaryotes. In comparison, prokaryotes have relatively few LHOs. The most contaminated assembly is *Pseudomonas aeruginosa* strain Pae221_ST274 (GenBank accession GCA_000825745.1) with 7 Kbp of human sequence.

**Fig 1:**
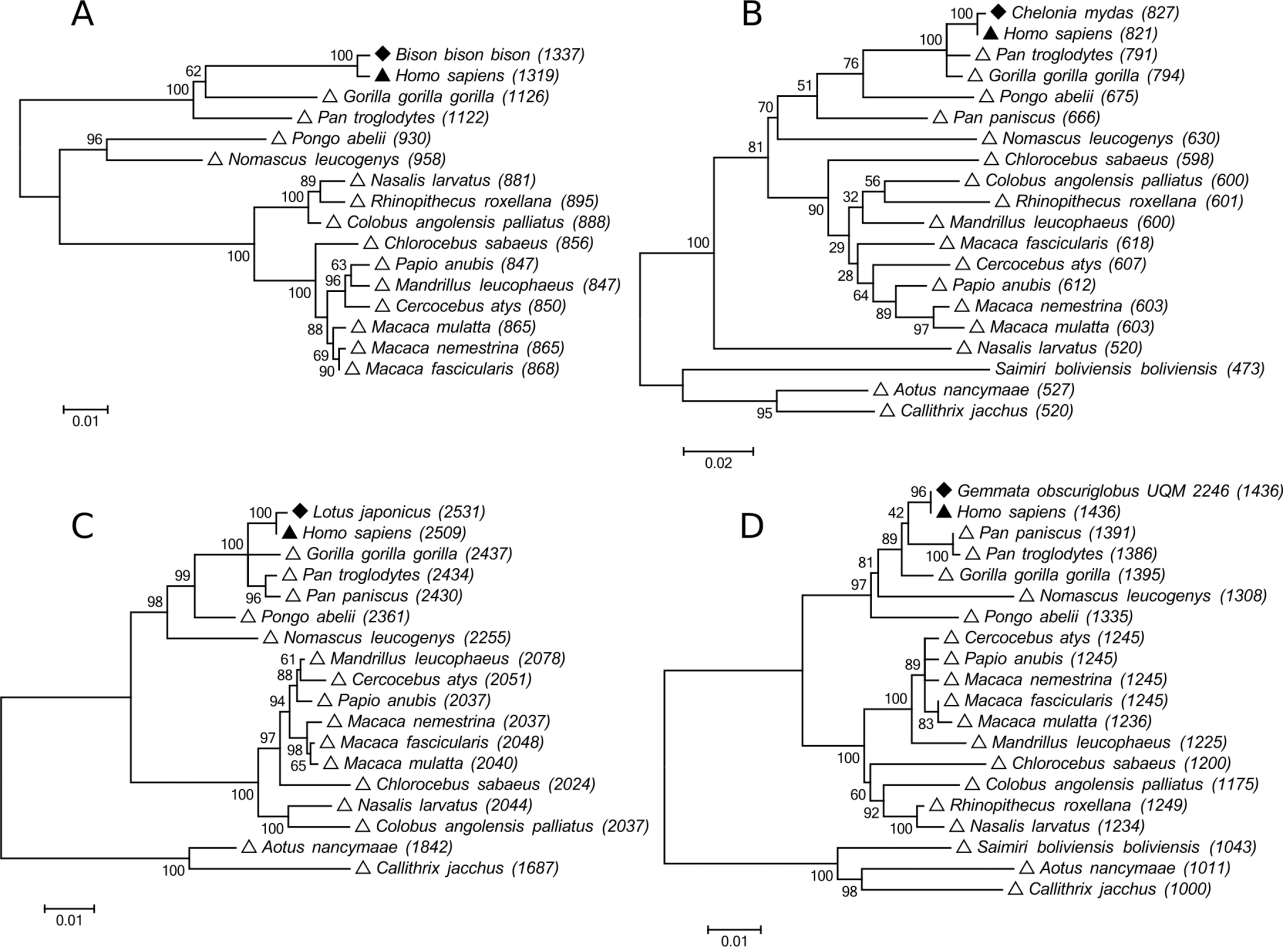
Phylogenetic trees comparing close homologs of human-originated regions.

In order to confirm that our method returns valid LHO sequences, we looked at the phylogenetic trees comparing selected LHO sequences with their closes homologs (Fig 1 and S1 Fig). We first selected the top LHO’s (those with highest primate specificity) in each of the four groups of genomes, extracted their homologs, multiply aligned, and built maximum likelihood trees (see Methods), shown on Fig 1. If the homology between the LHO sequence and human was due to conservation, we would expect the LHO sequence to be located outside of primates in the tree. Instead, in all four cases (Fig 1A–1D) LHO sequence is located deep within the primate cluster, and closest to human. This forces us to reject the conservation hypothesis, and consider alternative explanations: horizontal gene transfer and contamination. Among these two possibility, contamination from human seems much more likely scenario.

Phylogenetic trees comparing close homologs of the top most primate-specific human-originated regions found in each of the four groups of genomes: (A) mammals, (B) other vertebrates, (C) other eukaryotes, and (D) prokaryotes. Filled diamond marks the LHO sequence, filled triangle marks human, primates are marked by empty triangle. The numbers in parentheses are bit-scores of BLASTN hits with LHO as query.

In similar way we also examined the bottom LHO’s (those with smallest primate specificity) in each of the four groups of genomes, and performed the same steps of extracting homologs, aligning and building trees (S1 Fig). In these trees the resolving power of the phylogeny is smaller due to much shorter alignments, however close relationship between LHO and human is still apparent, as well as the location of LHO within primates. Examination of these trees allows us to conclude that our method detects valid LHO sequences.

**Fig 2:**
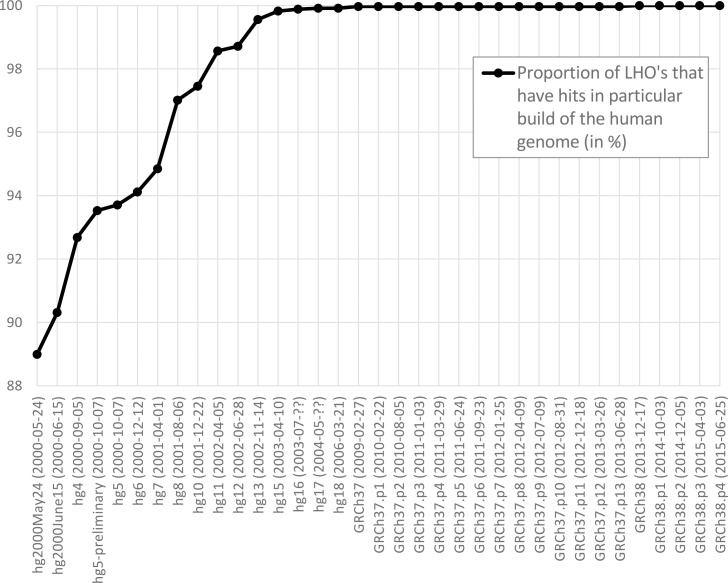
Proportion of LHO’s that have hits in older builds of the human genome.

Aiming to clarify the history of LHO sequence within the human genome, we counted how many LHO’s have homology to various builds of the human genome (Fig 2). Over 88% of LHO’s are already present in year 2000 draft of the genome. By the end of 2002 human genome assembly already incorporates over 99.5% of LHO’s. This means that the presence of human sequence in non-human genomes can’t be explained by the recent addition of the newly determined fragments. Already from 2002 a comparison with human genome assembly available at that time would have revealed strong homology that we see today.

We also checked how many of the LHO’s are in their own sequence within the assembly, as opposed to being a part of a long scaffold (S2 and S3 Figs). We found that relatively few LHO’s are found within much longer sequences, e.g., there are 104 LHO that constitute under 5% of the host sequences. The number of unassembled LHO’s is much larger: 357 of LHO’s (more than 10%) occupy over 99% of their host sequence, and 1,243 LHO’s (36%) occupy over a half of the length of their host sequence. Overall our observation suggests that LHO’s tend to remain unassembled and separate from other contigs.

## Discussion

In this study we detected sequences that are highly similar between human and remote organisms, including non-vertebrates. In theory such similarity can result from multiple scenarios, such as: (1) Genuine conservation. (2) Recent horizontal gene transfer. (3) Contamination in genome sequence. Our use of primate specificity score allows to separate real conservation from the remaining cases. Although we can’t completely rule out the possibility of horizontal gene transfer, such events are considered to be extremely rare in eukaryotes. On the other hand contamination is a known issue in sequencing experiments. Thus we conclude that most, if not all, of the LHO’s that we found are really contamination from human.

In the current study we only looked for the most undeniably human fragments in public genome assemblies. So we used a very strict criteria in our search: An LHO is a sequence with at least 95% nucleotide identity to repeat-masked human genome, containing no simple repeats, at least 100 bp long, and with primate specificity of at least 100 (200 for mammalian LHOs). This makes us reasonably confident that most of the LHOs we found are really human sequences, as we don’t expect our method to return many false positives.

Lowering the nucleotide identity threshold (95%), or minimum sequence length (100) might result in identifying larger number of potentially contaminating sequences. However in this study we decided to steer clear from investigation of limits of such analysis, and instead focus on clearly unambiguous examples of highly probably contamination. S1 Fig shows analysis of four example LHO regions with the lowest primate specificity among those we detected. It can be seen that even though the trees are less clear than in Fig 1, they still confidently support contamination / horizontal gene transfer scenario.

On the other hand, due to strictness of our method, we suspect that the actual number of human-originated sequences could be larger, and many such sequences are still undetected. For instance, repetitive sequence is just as likely to become contaminant as any other, however in this study we limited our scope to non-repetitive sequence to avoid discussing ambiguous cases. Some contamination can be masked by true conservation, especially in mammalian genomes. Some contamination has lower primate specificity than our detection threshold. Some human-originated sequence could be unique to humans, and thus missing in other primates, which would also make it undetectable with our method.

Also, while this study focuses only on human originated contamination, other sources of contamination exist, especially bacterial. In principle it is not difficult to extend this study to investigate other organisms as sources of contamination. However we limited our investigation to human due to the large computation time required for such analysis. Our analysis limited to human took about 1 month of calculation on a small cluster machine (16 nodes, 256 cores). In the future it may be interesting to apply the approach outlined in this work to other organisms.

One question naturally following from our results is whether the contamination sequences are recent addition to human genome. Initially we expected that LHO-producing sequences must be recent additions to human genome, and as such they were unavailable for comparison at the time of assembling the contaminated non-human genomes. However, to our surprise, the absolute majority (>99.5%) of LHO-producing human sequences were already present in hg13 build of the human genome, released in 2002. ~89% were already in the draft assembly from May 2000. This means that most of the LHO-producing human sequences are not a recent addition to the reference human genome assembly. Instead they have been there for over a decade. Therefore the strong similarity between LHO’s and the human genome could have been easily detected by a homology search. Normally a 100+ bp stretch of 95% nucleotide identity with remote organism should warrant close attention and careful treatment in assembly. The presence of such easily detectable LHO’s in multiple genome assemblies highlights the lack of general awareness about high possibility of contamination from human.

Genome sequences used as a reference is the basis for a multitude of biological, medical and environmental studies. Contamination present in a reference genome has potential to distort the results of such analyses. Therefore it is important to take all possible steps to avoid releasing genomes with easily detectable contamination. We suggest that new genome assembly projects should take extra care for avoiding human-originated contamination. Also we recommend that genome assemblies already present in public databases should be checked for human contamination and revised as necessary. In both cases the approach outlined in this study can be utilized.

For invertebrate genome assemblies, almost any long stretch of DNA that is highly homologous to human should be a suspect for contamination. However in case of vertebrate genome projects, true conservation with human may exist, such as human-mouse ultraconserved regions of over 200 bp and 100% identity. Therefore being able to separate contamination from conservation is important. This study shows how massive homology search can be used to find lineage specificity of a sequence, and how lineage specificity allows to separate true conservation from contamination. In this study we used primate specificity, because we focused on human contamination. However the same approach can be used with other contamination sources and their corresponding lineages.

## Methods

### Method overview

**Fig 3:**
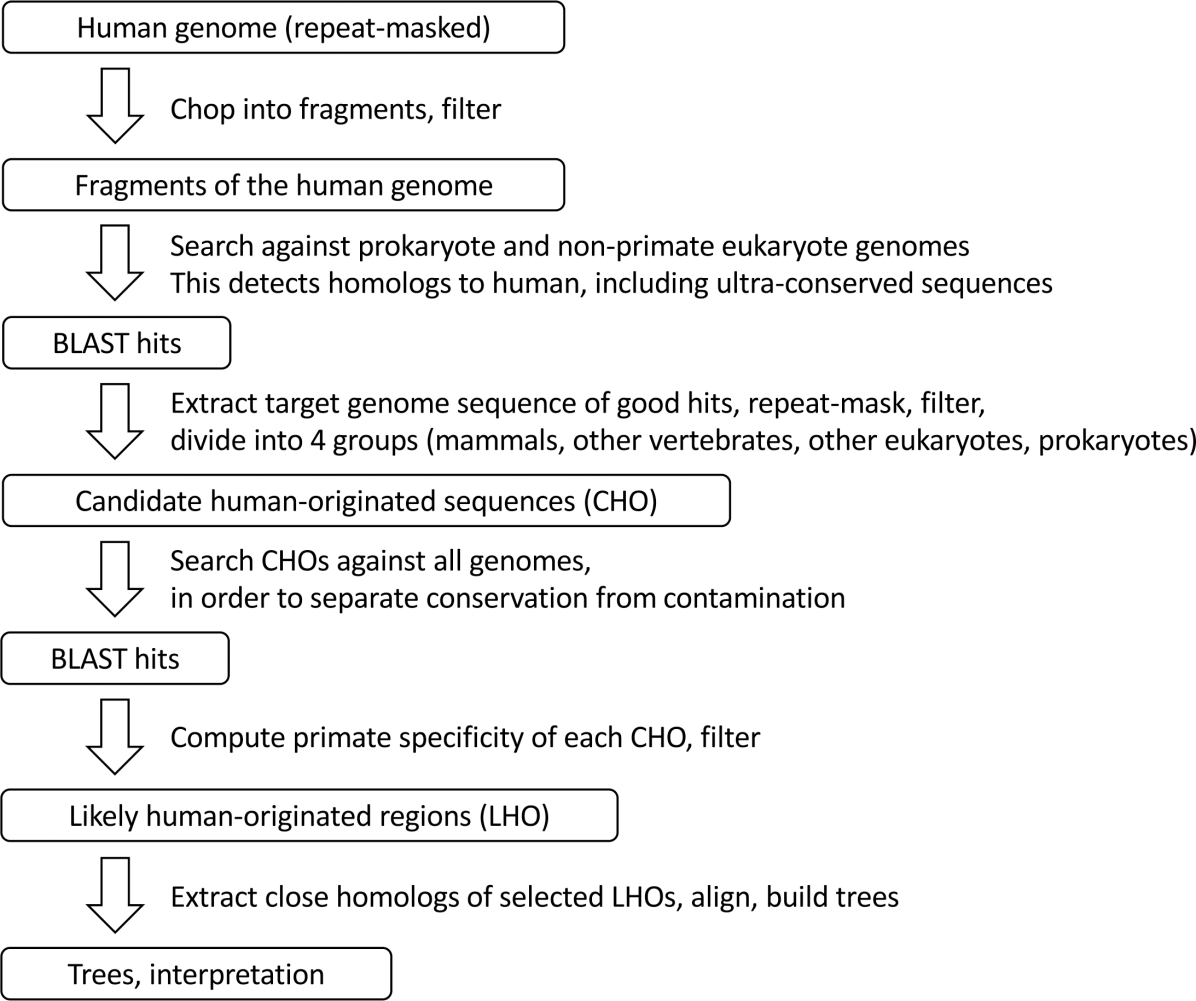
Schematic flowchart of the method.

Fig 3 shows the flowchart of our method. Essential steps of the procedure are the two homology searches. The first search locates homologs to human in other genomes. After filtering some of these homologs become CHOs (candidate human-originated sequences). Then the second search is conducted using CHOs as the query and all available genomes as reference, in order to clarify the origin of those CHOs. After the second search, CHOs that show specific homology to primates are chosen as LHO sequences.

### Human genome

We used the GRCh38.p4 build of the human genome (RefSeq accession: GCF_000001405.30), the latest at the time of starting analysis. We used all its sequences, including alternate loci and unplaced contigs, 510 sequences in total.

The historical builds of the human genome [8,9] used for investigating the LHO history were downloaded from UCSC [10], GenBank [11], and RefSeq [12]. In total we used 35 assemblies of human genome.

### Non-human genomes

We based our analysis on all genomes available at NCBI genome databases [11,12] on August 3, 2015, excluding redundant plant and animal genomes. Essentially we used all genomes that could be reasonably expected to serve as reference panel in sequence comparison, or in investigating unknown sequence. In total we used 50,602 genomes, with combined length of ~889 Gbp (Table 5). We divided the genomes into 6 groups, according to the NCBI taxonomy database [13]: Primates, other mammals, other vertebrates, other eukaryotes, prokaryotes, viruses.

**Table 5:** Summary of the genomes used.

Group	Genomes	Sequences	Total size (bp)
Primates	24	4,517,145	70,885,537,497
Non-primate mammals	82	9,207,268	210,526,504,865
Non-mammal vertebrates	133	13,837,380	146,702,887,277
Non-vertebrate eukaryotes	1,884	49,326,244	295,414,011,587
Prokaryotes	43,636	5,220,198	165,007,759,182
Viruses	4,843	6,379	189,550,904
Total	50,602	82,114,614	888,726,251,312

Accession numbers, dates and sizes of all used genomes are listed in S3 Dataset, containing the following files: “genome-list-primates.txt”, “genome-list-non-primate-mammals.txt”, “genome-list-non-mammal-vertebrates.txt”, “genome-list-non-vertebrate-eukaryotes.txt”, “genome-list-prokaryotes.txt”, and “genome-list-viruses.txt”.

We investigated 45,735 non-primate eukaryote and prokaryote genomes for signals of human-originated contamination, however all 50,602 genomes were used in the second search to investigate each possible contamination.

### First search

Initial search was aimed at finding human-like elements in non-primate genomes. We used the repeat-masked sequences of human genome. We hard-masked the sequences by replacing repetitive sequence with N. We then divided sequences at each continuous run of 10 or more N’s. This resulted in 11,191,562 fragments, with total length of 1,822,301,504 bp, which were used as a query.

Since we were looking for sequence highly homologous to human, we used BLAST search [14] with the command “blastn -task megablast -evalue 1e-5 -dbsize 3200000000”. The database consisted of 45,735 non-primate genomes (2,099 eukaryote and 43,636 prokaryote), with a total sequence size of 817,651,162,911 bp. The search produced 63,358,441 hits.

### Extracting and filtering the regions

We extracted all hits with identity of at least 95%, obtaining 3,891,658 sequences. We then masked the repetitive sequence using DUST [15] with command “dustmasker–level 30”. We then kept only continuous non-repetitive fragments of at least 100 bp. This resulted in 1,010,402 candidate human-originated sequences (CHO).

### Second search

We performed the second search using CHO’s as queries. All available genomes (50,602 genomes, ~889 Gbp) were used as database. We used BLASTN with “-evalue 3.80e-2 -dbsize 3200000000” for non-mammalian CHO’s, and MEGABLAST with “-evalue 1e-5 -dbsize 3200000000” for mammalian CHO’s. When a CHO had multiple hits in particular genome, we preserved only the best hit.

### Primate specificity

We looked at each of the CHO sequences, and computed two numbers: S_p_ is the score of the best hit within primates, and S_np_ is the score of the best hit outside of primates. We then computed primate specificity (PS) as the difference S_p_−S_np_. High primate specificity indicates low chance for conservation and makes contamination a most likely explanation. We set PS threshold of 100 for non-mammals and 200 for mammals. To minimize the chance of false positives, we also removed all CHO’s with human hit score lower than 98% of the best score in primates, and those with human hit score lower than 150% of self-hit score. The resulting sequences are likely human originated (LHO).

### Phylogeny

To verify that the LHO’s match our expectation, we conducted phylogenetic reconstruction for a selected subset of the LHO’s. To do that, for a given LHO, we extracted all its homologs having score of at least S/2, where S is the score of self-hit (using at most one hit per genome). We then multiply aligned the whole set of homologs, together with the LHO, using MAFFT [16] with “—localpair—maxiterate 1000”. We then constructed maximum likelihood tree in MEGA6 [17].

### Comparing with older builds of human genomes

The homology search was run using command: “blastn -task blastn -evalue 3.80e-2 -dbsize 3200000000 -outfmt 6”, for each of the 35 assemblies of human genome. After that we counted how many LHO’s have hits in each search.

## Supporting Information

S1 Dataset LHO Sequences. (ZIP)

S2 Dataset LHO bed files. (ZIP)

S3 Dataset Genome lists. (ZIP)

S1 Fig Phylogenetic trees comparing close homologs of the bottom (least primate-specific) human-originated regions found in each of the four genome groups. (A) mammals, (B) other vertebrates, (C) other eukaryotes, and (D) prokaryotes. Filled diamond marks LHO sequence, filled triangle marks human, empty triangle marks primates, empty diamonds marks genomes that are closer to LHO-containing organism than to human in taxonomy. The numbers in parentheses are bit-scores of BLASTN hits with LHO as query.
(PDF)

S2 Fig Histogram of relative LHO sizes, in percents from the sizes of genomic contigs or scaffolds harboring them, binned with the resolution of 1%. (PDF)

S3 Fig Cumulative chart of relative LHO sizes. (PDF)

S1 Table Non-primate mammalian genomes containing likely human originated (LHO) sequence. (XLSX)

S2 Table Non-mammal vertebrate genomes containing likely human originated (LHO) sequence. (XLSX)

S3 Table Non-vertebrate eukaryote genomes containing likely human originated (LHO) sequence. (XLSX)

S4 Table Prokaryote genomes containing likely human originated (LHO) sequence. (XLSX)
