# Supplementary material for: Human Contamination in Public Genome Assemblies
Source: PLoS One. 2016 Sep 9;11(9):e0162424. doi: 10.1371/journal.pone.0162424 (PMC5017631; doi:10.1371/journal.pone.0162424)
Supplement: S1 Fig — (A) mammals, (B) other vertebrates, (C) other eukaryotes, and (D) prokaryotes. Filled diamond marks LHO sequence, filled triangle marks human, empty triangle marks primates, empty diamonds marks genomes that are closer to LHO-containing organism than to human in taxonomy. The numbers in parentheses are bit-scores of BLASTN hits with LHO as query. (PDF) [file pone.0162424.s004.pdf]

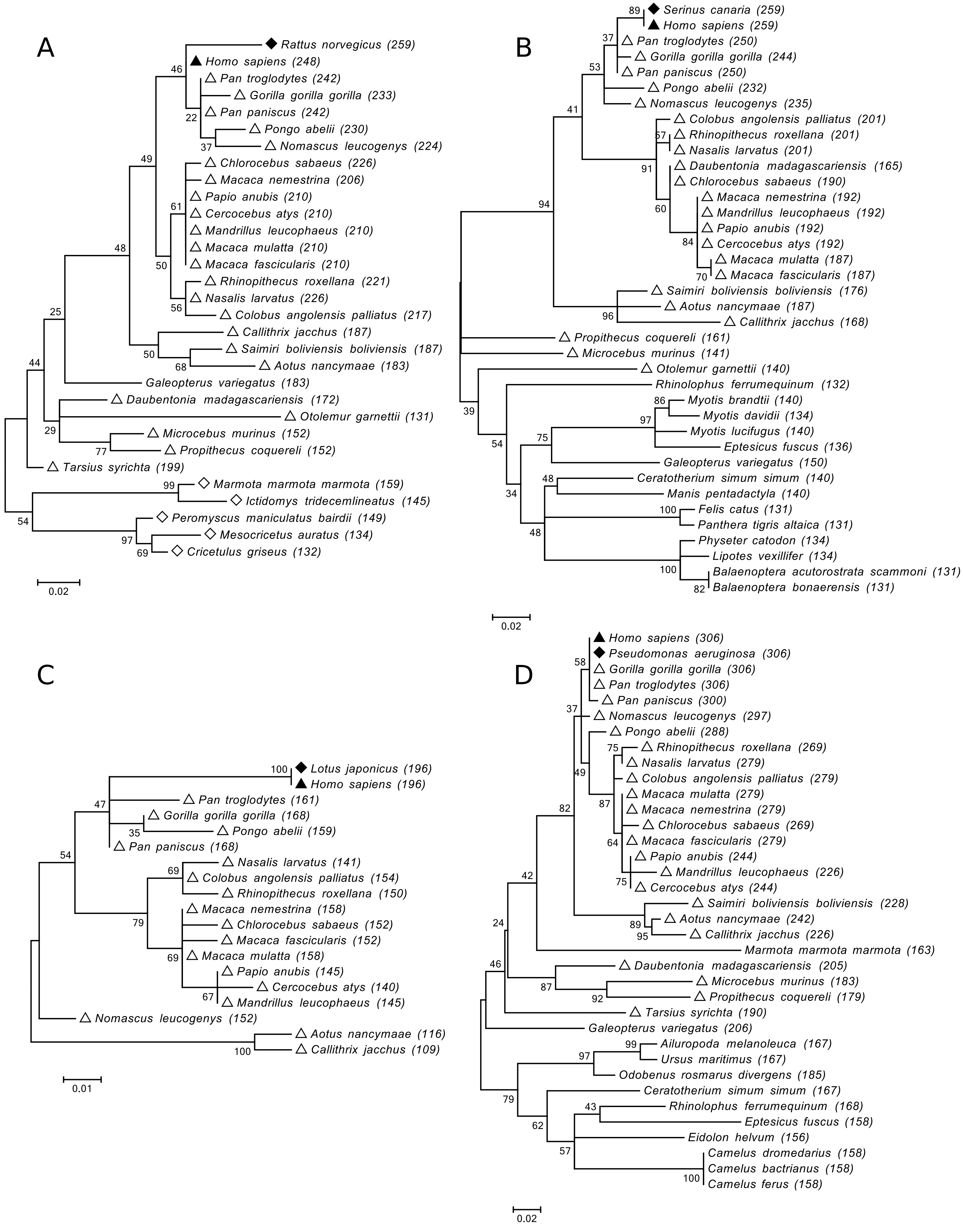

S1 Fig. Phylogenetic trees comparing close homologs of the bottom (least primate-specific) human-originated regions found in each of the four genome groups. (A) mammals, (B) other vertebrates, (C) other eukaryotes, and (D) prokaryotes. Filled diamond marks LHO sequence, filled triangle marks human, empty triangle marks primates, empty diamonds marks genomes that are closer to LHO-containing organism than to human in taxonomy. The numbers in parentheses are bit-scores of BLASTN hits with LHO as query.
