# Supplementary material for: Human Contamination in Public Genome Assemblies
Source: PLoS One. 2016 Sep 9;11(9):e0162424. doi: 10.1371/journal.pone.0162424 (PMC5017631; doi:10.1371/journal.pone.0162424)
Supplement: S2 Fig — (PDF) [file pone.0162424.s005.pdf]

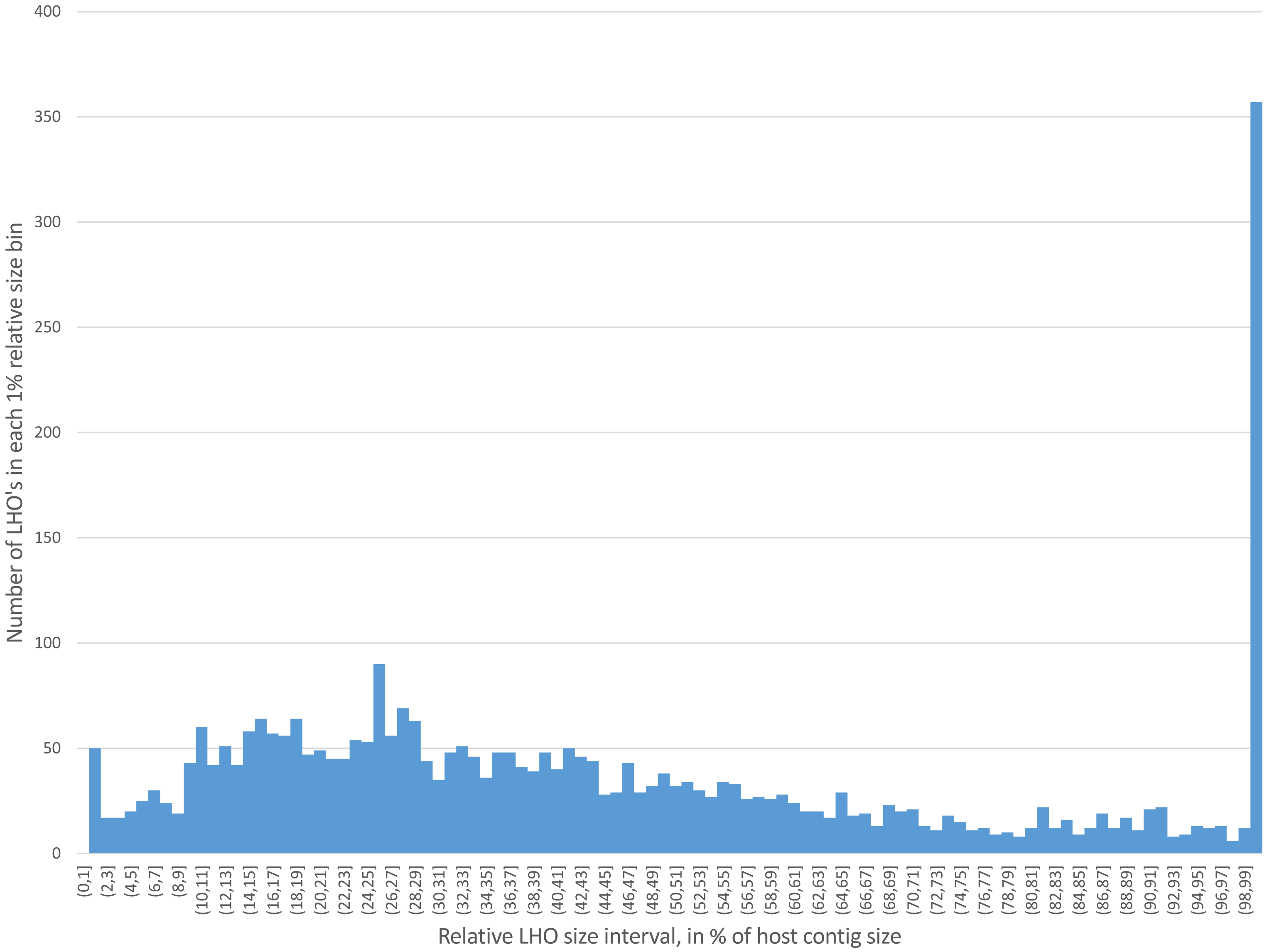

S2 Fig. Histogram of relative LHO sizes, in percents from the sizes of genomic contigs or scaffolds harboring them, binned with the resolution of 1%.
