# Supplementary figures and images for: Human Contamination in Public Genome Assemblies
Source: PLoS One. 2016 Sep 9;11(9):e0162424. doi: 10.1371/journal.pone.0162424 (PMC5017631; doi:10.1371/journal.pone.0162424)

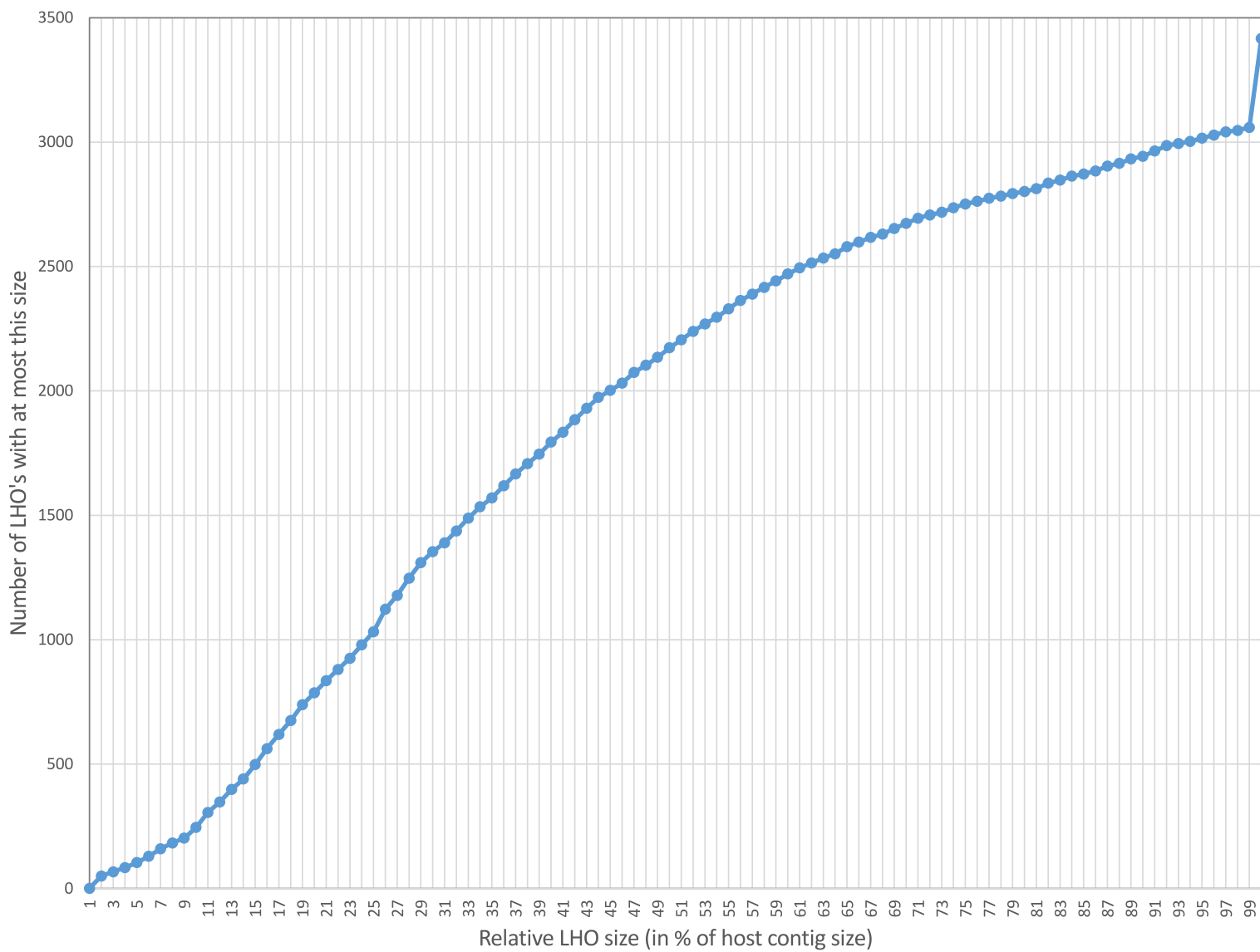

**S3 Fig.** Cumulative chart of relative LHO sizes.

Supplement: S3 Fig — (PDF) [file pone.0162424.s006.pdf]
